# Development and initial characterization of a high-resolution PET detector module with DOI

**DOI:** 10.1088/2057-1976/abbd4f

**Published:** 2020-10-14

**Authors:** Mohan Li, Yuli Wang, Shiva Abbaszadeh

**Affiliations:** 1 Department of Nuclear, Plasma, and Radiological Engineering, University of Illinois at Urbana-Champaign, Urbana, IL, 61801, United States of America; 2 Department of Electrical and Computer Engineering, University of California, Santa Cruz, Santa Cruz, 95064, United States of America

**Keywords:** depth of interaction, lutetium-yttrium oxyorthosilicate (LYSO), silicon photomultipliers (SiPM), positron emission tomography, time, energy and spatial resolution

## Abstract

Organ-dedicated PET scanners are becoming more prevalent because of their advantages in higher sensitivity, improved image quality, and lower cost. Detectors utilized in these scanners have finer pixel size with depth of interaction (DOI) capability. This work presents a LYSO(Ce) detector module with DOI capability which has the potential to be scaled up to a high-resolution small animal or organ-dedicated PET system. For DOI capability, a submodule with one LYSO block detector utilizing PETsys TOFPET2 application-specific integrated circuit (ASIC) was previously developed in our lab. We scaled up the submodule and optimized the configuration to allow for a compact housing of the dual-readout boards in one side of the blocks by designing a high-speed dual-readout cable to maintain the original pin-to-pin relationship between the Samtec connectors. The module size is 53.8 × 57.8 mm^2^. Each module has 2 × 2 LYSO blocks, each LYSO block consists of 4 × 4 LYSO units, and each LYSO unit contains a 6 × 6 array of 1 × 1 × 20 mm^3^ LYSO crystals. The four lateral surfaces of LYSO crystal were mechanically ground to W14, and the two end surfaces were polished. Two ends of the LYSO crystal are optically connected to SiPM for DOI measurement. Eight LYSO blocks performance including energy, timing, and DOI resolution is characterized with a single LYSO slab. The in-panel and orthogonal-panel spatial resolution of the two modules with 107.4 mm distance between each other are measured at 9 positions within the field of view (FOV) with a ^22^Na source. Results show that the average energy, timing, and DOI resolution of all LYSO blocks are 16.13% ± 1.01% at 511 keV, 658.03 ± 15.18 ps, and 2.62 ± 0.06 mm, respectively. The energy and timing resolution of two modules are 16.35% and 0.86 ns, respectively. The in-panel and orthogonal-panel spatial resolution of the two modules at the FOV center are 1.9 and 4.4 mm respectively.

## 1. Introduction

Organ-dedicated positron emission tomography (PET) scanners can provide improved spatial resolution by using finer scintillator crystals, higher system sensitivity by placing the detectors closer to the organ, better image contrast recovery by reducing the noise from other organs, and lower cost by reducing scanner geometry (González *et al* 2018). To adapt to different imaging environment and offer the flexibility to place the detectors around the organ, limited angle geometry instead of complete ring-shaped geometry is utilized in systems dedicating to breast (MacDonald *et al* 2009, Raylman 2009), heart (Peng 2015), and prostate (Zorraquino *et al* 2013).

The measurement of the DOI is of critical importance for a PET scanner with compact geometry to simultaneously achieve high and uniform spatial resolution, and high photon sensitivity (Bailey *et al* 2003, Yang *et al* 2016). Developing a practical DOI-capable PET detector with balanced performance has been studied for over two decades, including but not limited to dual-ended readout method using two detectors coupled to the both ends of crystal (Moses and Derenzo 1994, Miyaoka *et al* 1998, Yang *et al* 2006, Ling *et al* 2007, Maas *et al* 2009), single-ended readout method using one detector coupled to one end of crystal (Berg *et al* 2016), side readout method by reading signal from crystal lateral surface (Yamaya *et al* 2006, Yeom *et al* 2014), light-sharing window-based method (Zhang *et al* 2019), monolithic scintillator detector based method (Schaart *et al* 2009), single-ended readout method with light guide on the top surface (Pizzichemi *et al* 2016). Among all DOI methods, dual-ended readout method has the advantages of a continuous DOI measurement and a balanced detector performance by increasing the total light output (Shao *et al* 2002, Burr *et al* 2004, Yang *et al* 2008, Kuang *et al* 2017).

The selection of photon sensors and their proper coupling to scintillators has a relatively large impact on the performance of final PET system. The conventional photomultiplier tube (PMT) is bulky and has a large insensitive area, and the gain across the photo-cathode of a PMT is non-uniform. The avalanche photodiode (APD) has a relatively large insensitive area at the edge and an intrinsically low signal-to-noise ratio (Yang *et al* 2008). Over the last few years, silicon photomultiplier (SiPM) has advanced tremendously in terms of cost and performance. Compared with PMT and APD, SiPM offers high amplification gain, compact size, small insensitive area, and low voltage bias, and has been widely used for dual-ended readout design (Herbert *et al* 2006, Schaart *et al* 2009, Yamaya *et al* 2011, Nadig *et al* 2019).

Our lab has developed a high-resolution PET detector submodule with dual-ended readout capability based on SiPMs and PETsys TOFPET2 application-specific integrated circuits (ASIC), which allows us to read out each SiPM channel independently (Li and Abbaszadeh 2019). In this study, the submodule is completed and configured to a full module that can be further scaled up to build high-resolution PET systems with DOI capabilities. To make the geometry compact, we optimized the previous lutetium-yttrium oxyorthosilicate (LYSO) block detector configuration by designing a high-speed dual-readout cable that accommodates the readout electronics on the same side of the scintillator. The energy, timing, and DOI resolution of eight detector blocks with dual-readout cable are characterized and are compared with previous design (Li and Abbaszadeh 2019). Four detector blocks formed a detector module with area of 53.8 × 57.8 mm^2^ and the energy and time resolution of the two detector modules were measured at 107.4 mm distance from each other. Spatial resolution at different positions within this FOV was measured.

## 2. Materials and methods

### 2.1. Detector module

**Figure 1.**
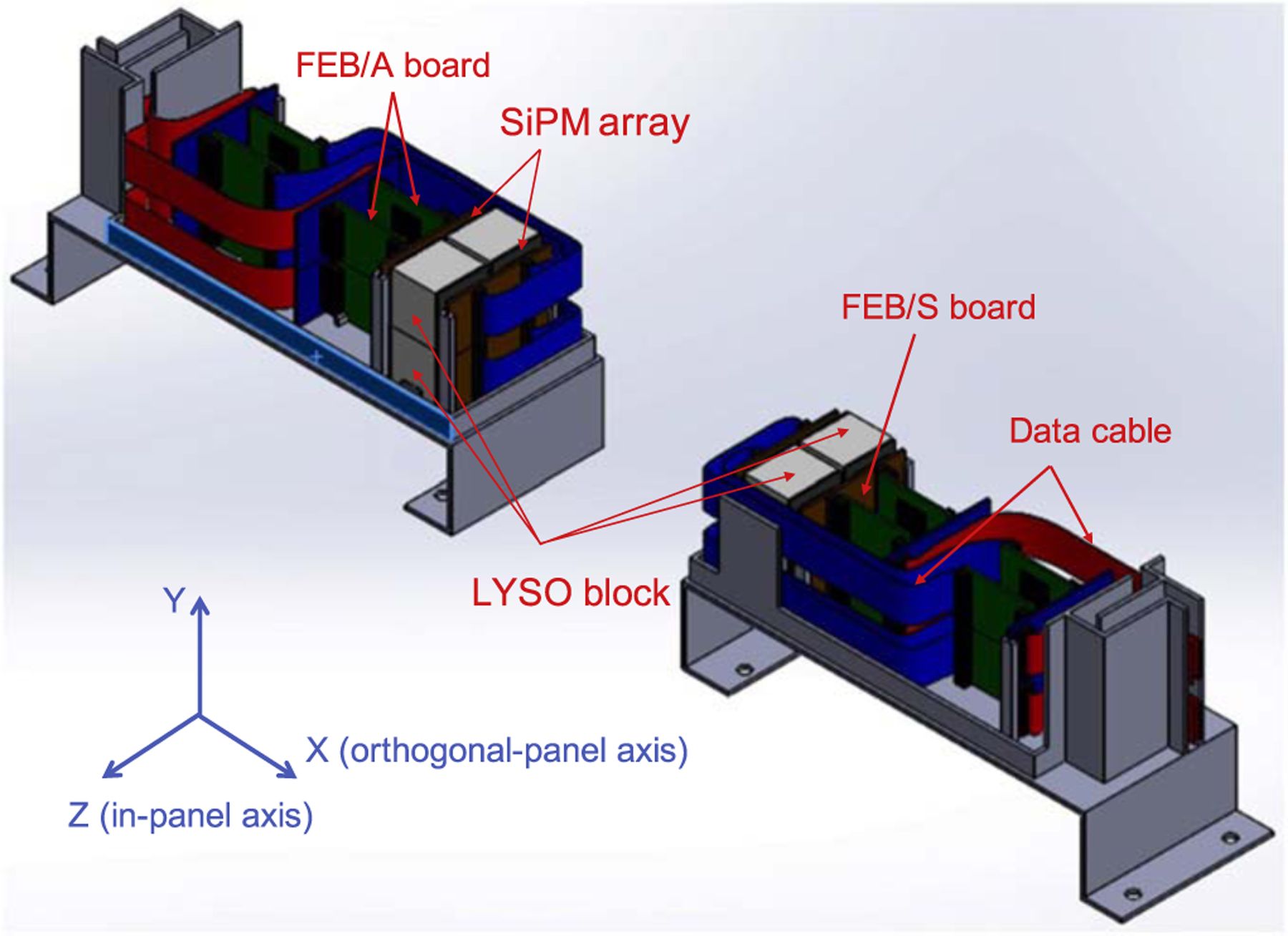
The schematic design of the detector module, containing LYSO blocks, SiPM, arrays, TOFPET2 ASIC readout electronics and data transmitted cable.

The schematic design of the detector module is shown in figure 1. A module has 2 × 2 LYSO blocks. The details of LYSO block fabrication is shown in (Li and Abbaszadeh 2019). The analog output signals of the SiPMs were digitized by PETsys TOFPET2 ASIC (PETsys Electronics SA, Portugal) and acquired by PETsys SiPM Readout System. When making the detector module with dual-ended readout capability, the electronics would take space within the FOV and increase the panel distance of the detector module. To make the geometry compact, it can be seen that the TOFPET2 ASIC electronics were placed on one side of the LYSO block.

**Figure 2.**
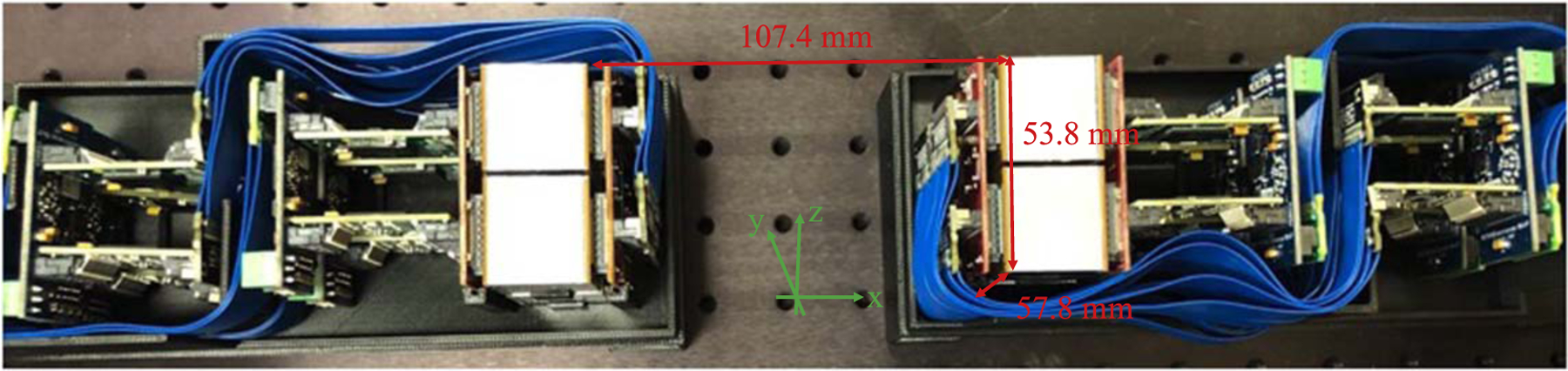
The picture of two detector modules.

Figure 2 shows the picture of two detector modules developed in this work separated 107.4 mm from each other. The TOFPET2 ASICs and SiPM arrays of the detector module were cooled by a Peltier element (CUI Devices CP455535H) and a heat sink (Advanced Thermal Solutions ATS-52450P-C1-R0). The Peltier element was attached to a 3D printed lid with a thermally conductive adhesive (3M Electronic Specialty TC-2810-50ML), and the heat sink is on the top of the Peltier element. The whole lid was placed on the top of the detector module to keep the environment temperature around the detectors stable and to shield from environment light.

**Figure 3.**
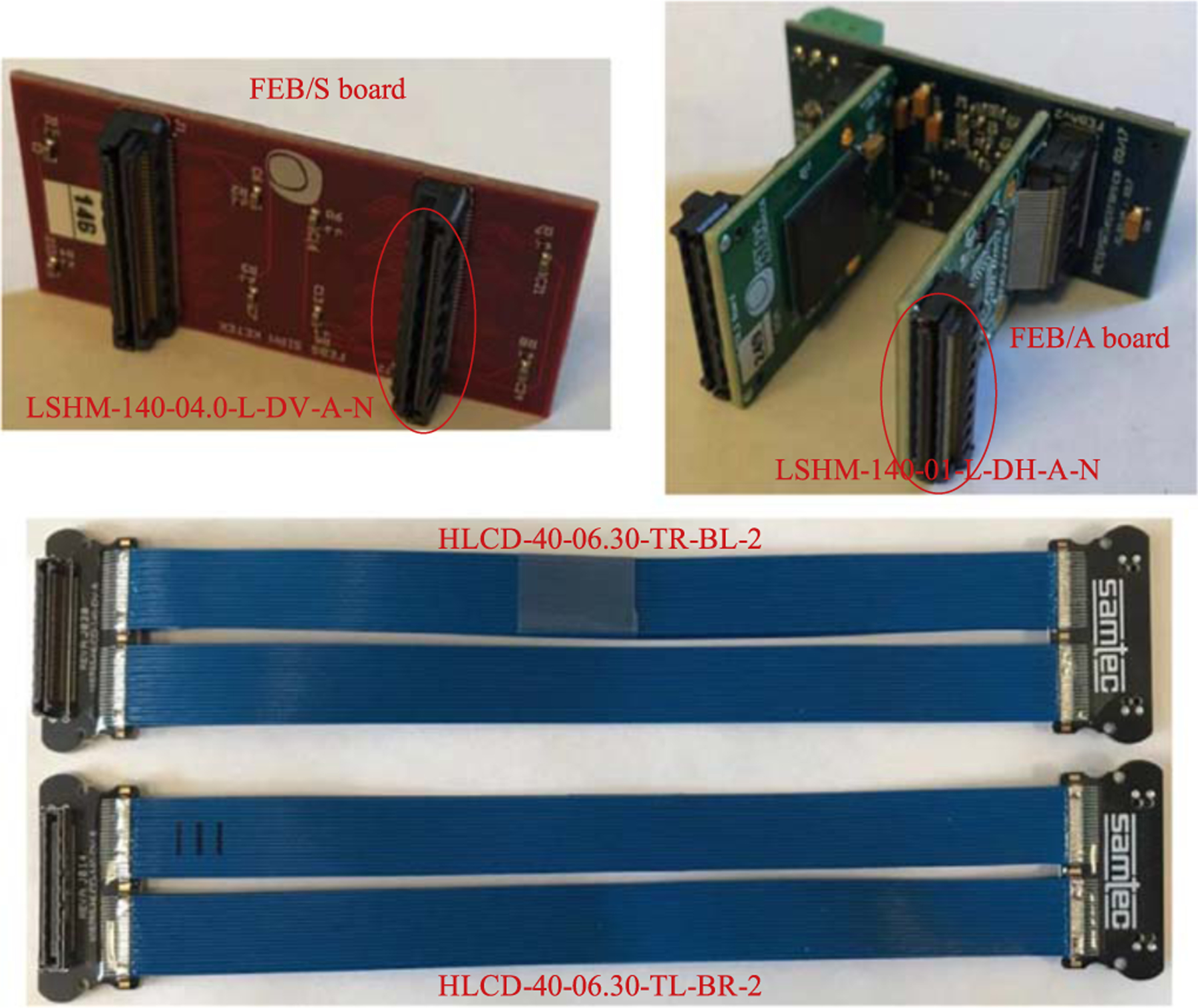
Top left: PETsys FEB/S board has two Samtec LSHM-140-04.0-L-DV-A-N connectors. Top right: PETsys FEB/A board has a Samtec LSHM-140-01-L-DH-A-N connector. Bottom: the picture of Samtc HLCD-40-06.30-TR-BL-2 and HLCD-40-06.30-TL-BR-2 cables.

Figure 3 shows the specific components of TOFPET2 ASIC readout electronics, including PETsys FEB/S board and FEB/A board. FEB/S was the board coupled to SiPMs, and FEB/A was the board hosting the TOFPET2 ASIC. Both FEB/S and FEB/A boards have two LSHM connectors (Samtec Inc., US) for connecting. In order to keep FEB/S board connected to SiPM and both FEB/A boards on one side of LYSO blocks, we designed a dual-readout cable. Due to the performance advantages in high speed, wide bandwidth, small signal loss and small crosstalk, Samtec HLCD cables were used as the data cables in this application which host two LSHM connectors. Depending on the geometry requirement and the original pin-to-pin relationship, HLCD-40-06.30-TR-BL-2 and HLCD-40-06.30-TL-BR-2 (Samtec Inc., US) were chosen for this application. The picture of the two custom-made cables is shown in figure 3 bottom.

### 2.2. Flood histogram of LYSO unit

To characterize the detector module, the flood histogram of each LYSO unit was measured as a first step. As described in section 2.1, the PETsys TOFPET2 ASIC and PETsys SiPM Readout System were used to digitize and acquire the analog output signals from SiPM arrays boards. A 30-*μCi* Na-22 source (Eckert & Ziegler Inc., Germany) with an active diameter of 0.25 mm was used to irradiate the LYSO unit. During the experiments, the timing output and energy output would be read out and recorded, when a SiPM channel get triggered.

**Figure 4.**
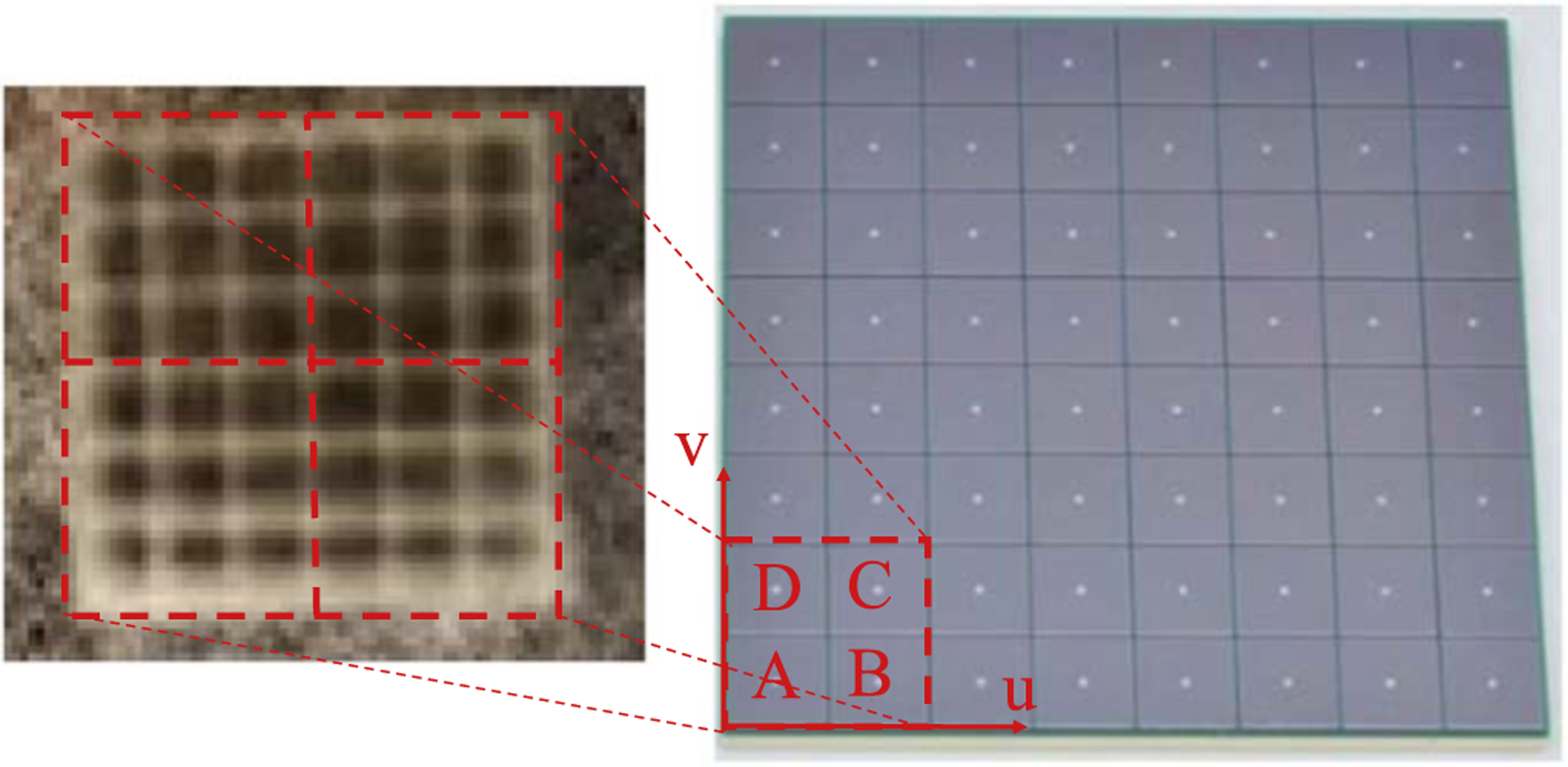
Different LYSO units are read out independently, and one LYSO unit is read out by two 2 × 2 SiPM arrays from both ends.

Figure 4 shows the flood histogram coordinate definition of one LYSO unit. The flood histogram was calculated using the position-encoding energy signals from the two 2 × 2 SiPM arrays as discussed in (Ren *et al* 2014),

(1) $$u=\frac{1}{2}\left(\frac{B_{1}+C_{1}}{E_{1}}+\frac{B_{2}+C_{2}}{E_{2}}\right),\;v=\frac{1}{2}\left(\frac{C_{1}+D_{1}}{E_{1}}+\frac{C_{2}+D_{2}}{E_{2}}\right),$$

where *A*_1_, *B*_1_, *C*_1_ and *D*_1_ are the four energy outputs from the SiPM array on the one end of the LYSO unit and *A*_2_, *B*_2_, *C*_2_ and *D*_2_ are from the SiPM array on the other end. *E*_1_ and *E*_2_ are the total energy measured by the two SiPM arrays respectively as

(2) $$E_{1}=A_{1}+B_{1}+C_{1}+D_{1},\;E_{2}=A_{2}+B_{2}+C_{2}+D_{2}.$$

### 2.3. Energy, time and DOI resolution of LYSO block

**Figure 5.**
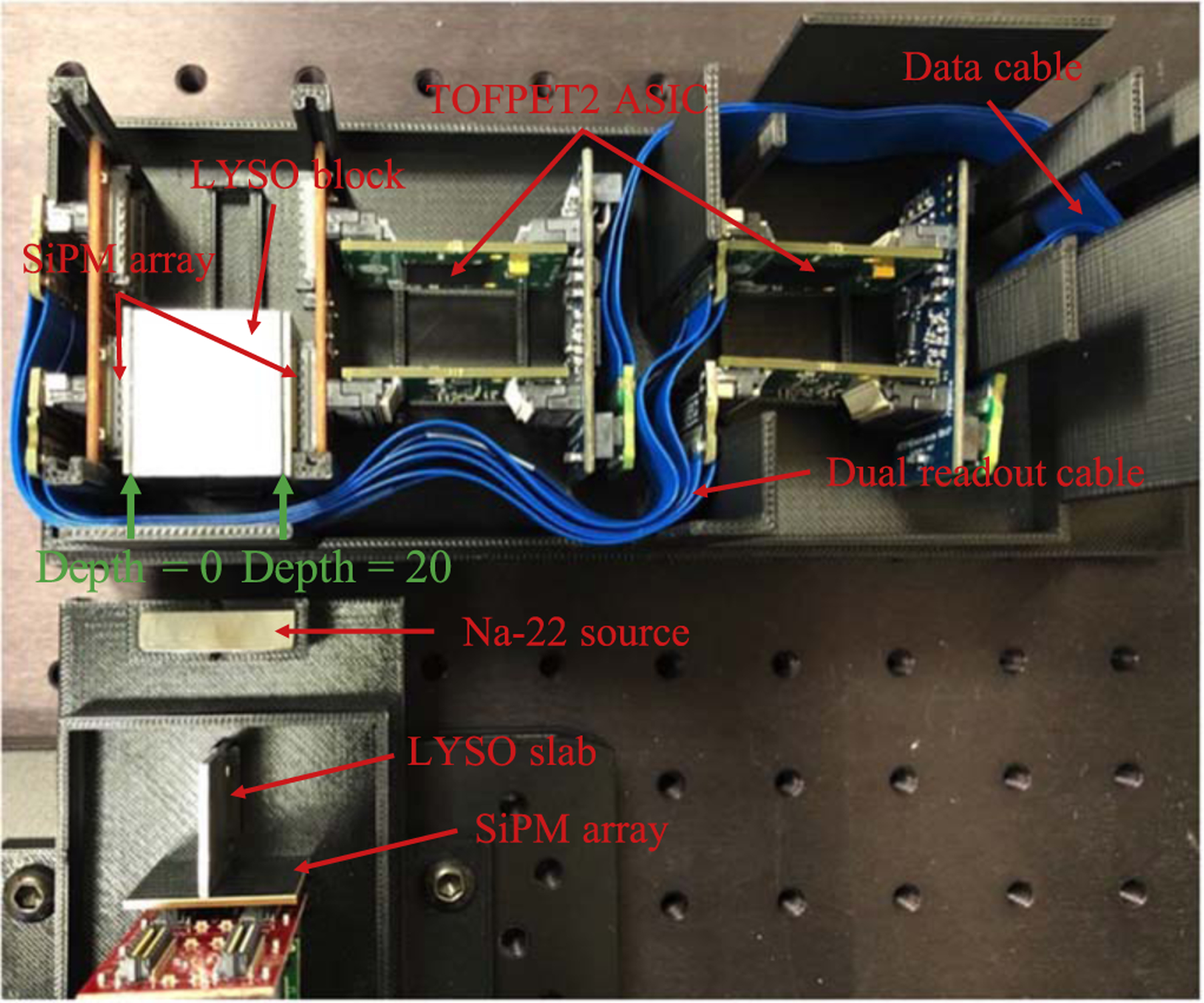
The experiment setup for characterizing the LYSO block performance, including energy, timing and DOI resolution.

A single 1 × 25.8 × 20 mm^3^ LYSO slab was used to characterize the energy resolution, timing resolution and DOI resolution of designed LYSO block by the experimental setup shown in figure 5. The LYSO slab was also read out by the same SiPM arrays as the LYSO block, but with only 8 channels aligned in a column. The same Na-22 source was used, located between the LYSO block and LYSO slab and had 20 mm distance from each of them. The Na-22 source and LYSO slab were on a translation stage, which could move along the depth direction of the LYSO block. The incident beam width was 1 mm in this experiment, which was determined by the width of the LYSO slab.

All 8 LYSO blocks within the two detector modules were measured individually by the experimental setup shown in figure 5. For the LYSO block, the side read out by the dual-readout cable is defined as depth 0 mm, and the side being directly read out is defined as depth 20 mm. Data were acquired at 5 depths (2, 6, 10, 14, 18 mm) for each detector, and each depth had a 5 min acquisition time. The SiPMs and ASICs were cooled down by a Peltier element and a fan. During the experiment, the SiPM temperature was maintained at 24.3 ± 1.9 °C.

The energy resolution (E) of an event was measured as

(3) $$E=E_{1}+E_{2}.$$

The coincidence time (t) was estimated as

(4) $$t=\text{min}\left(t_{1},t_{2}\ldots ,t_{8}\right)-\text{min}\left(t_{9},t_{10},\ldots ,t_{16}\right),$$

where *t*_1_ to *t*_8_ are the earliest triggered timing outputs of 8 SiPM channels (corresponding to one LYSO unit) out of the 128 channels in a LYSO block (one block consists of 4 × 4 LYSO units), and *t*_9_ to *t*_16_ are the earliest triggered timing outputs of the of the LYSO slab. The DOI ratio was estimated as

(5) $$DOI\;\text{ratio}=\frac{E_{1}}{E_{1}+E_{2}}.$$

A DOI calibration curve was used to convert the DOI ratio to the interaction depth (presented in figure 9), which was obtained by a linear fit of the peak value of the DOI ratio histogram to the known depth of interaction (interaction depth = a × DOI ratio + b). The DOI resolution was acquired by a Gaussian fit of the depth histogram.

### 2.4. Spatial resolution

After completing the characteristics measuring of each LYSO block, all 8 LYSO blocks were assembled to two detector modules. The energy and timing resolution of the modules were characterized at 104.7 mm distance from each other. The spatial resolution within the FOV was calibrated by a Na-22 source. The spatial resolution was measured at the FOV center and 5, 10, 15, 20 mm away from the center along the in-panel (*z* axis) and orthogonal-panel (*x* axis) directions. Each source position had 300-s data acquisition time, and the temperature was controlled as 32.2 ± 0.9 °C. The increase in the temperature is due to operation of 4 LYSO blocks in the module with the same Peltier element as opposed to one LYSO block in the setup shown in figure 5.

## 3. Results and discussion

### 3.1. Flood histogram of LYSO unit

**Figure 6.**
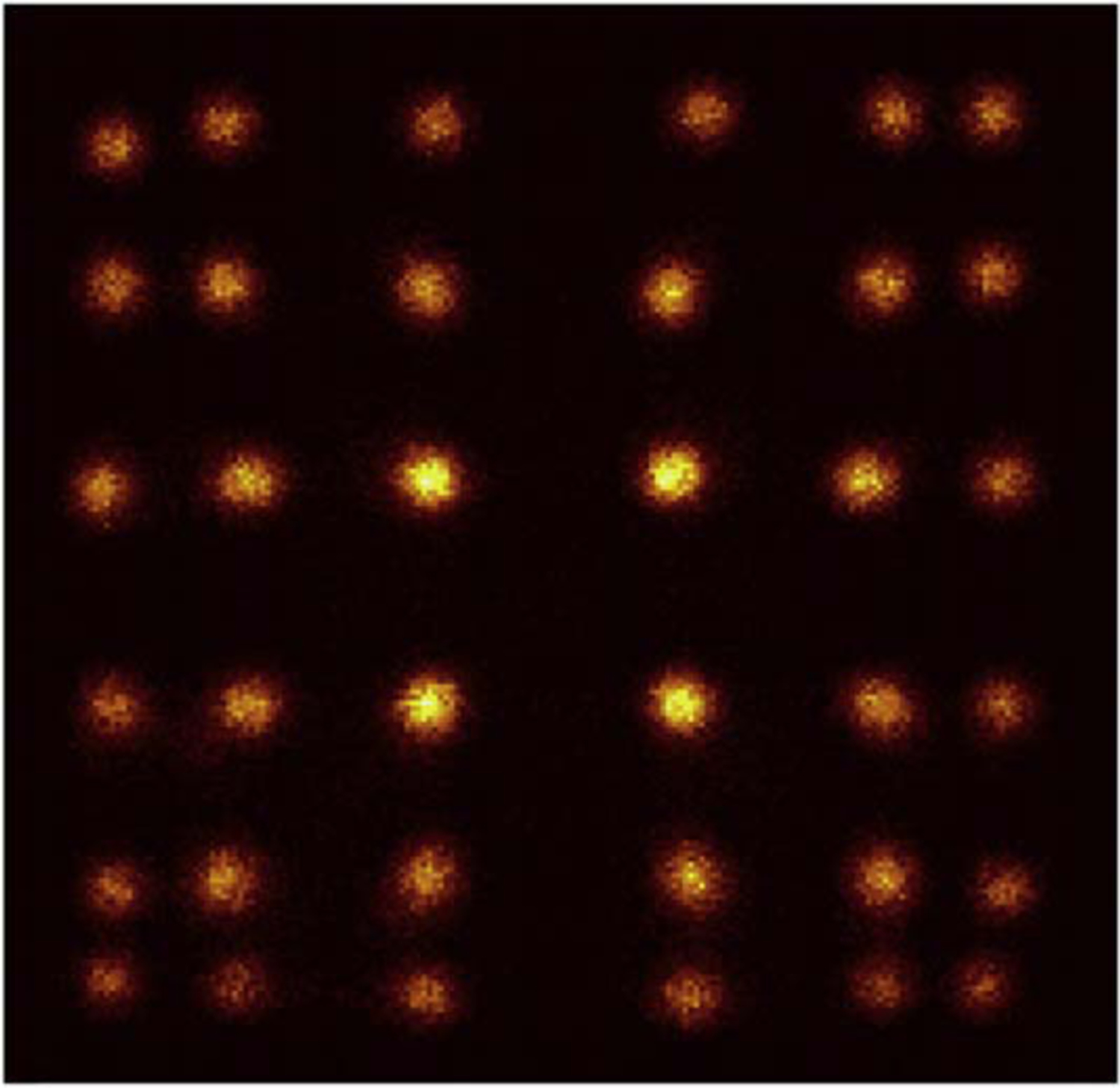
An example of the flood histogram of one LYSO unit.

An example of the flood histogram of one LYSO unit is shown figure 6. All the 6×6 array of LYSO crystals within one LYSO unit could be resolved. The center LYSO crystals were brighter than the LYSO crystals near the sides, which indicates a higher detection efficiency in the center LYSO crystal. We described more details about crystal resolvability, light guide thickness, and how the flood histogram was obtained in (Li and Abbaszadeh 2019).

### 3.2. Energy, timing and DOI resolution of LYSO blocks

**Figure 7.**
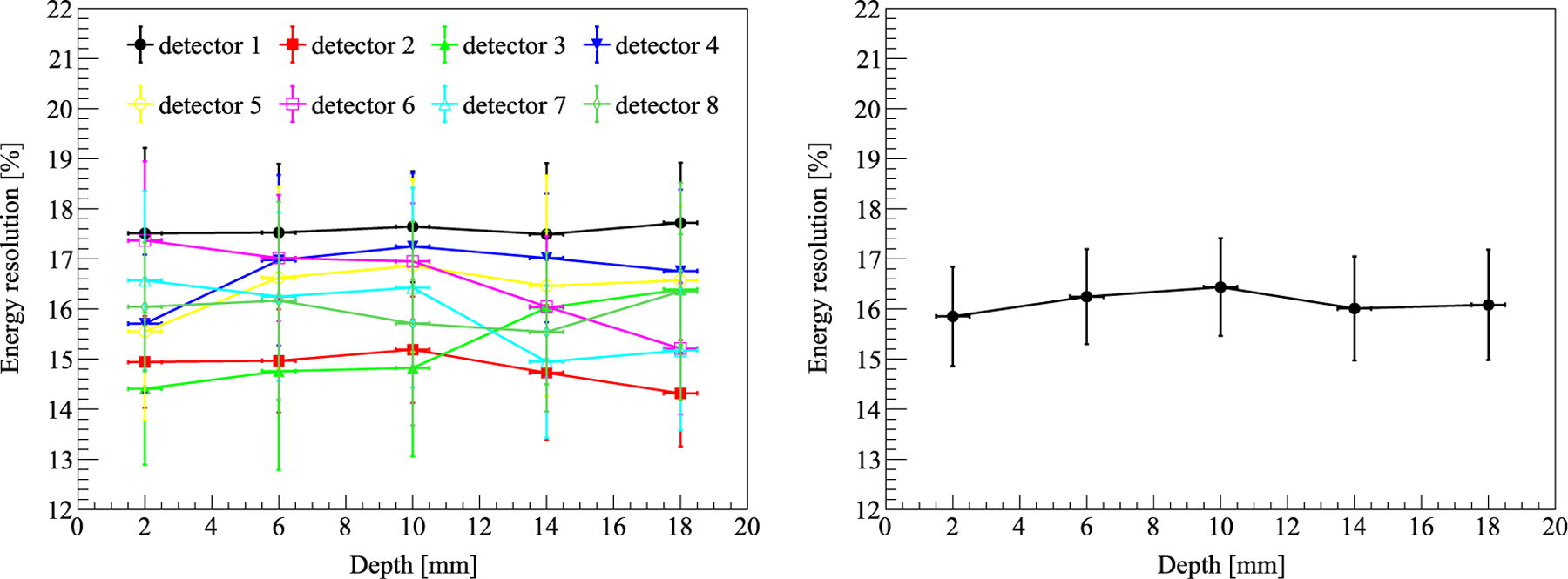
Left: the energy resolution of all 8 LYSO blocks at 5 depths. The error bar is the standard deviation of 16 LYSO units in each detector. Right: the average energy resolution of all 8 LYSO blocks. The error bar is the standard deviation of the 8 LYSO blocks at a specific depth.

The energy resolution of all 8 LYSO blocks is shown in figure 7. As shown in figure 7 left, the energy resolution did not show an obvious dependence on the interaction depth over all LYSO blocks. Based on results of figure 7 right, the average energy resolution of all 8 LYSO blocks at all 5 depths was 16.13% ± 1.01% at 511 keV.

**Figure 8.**
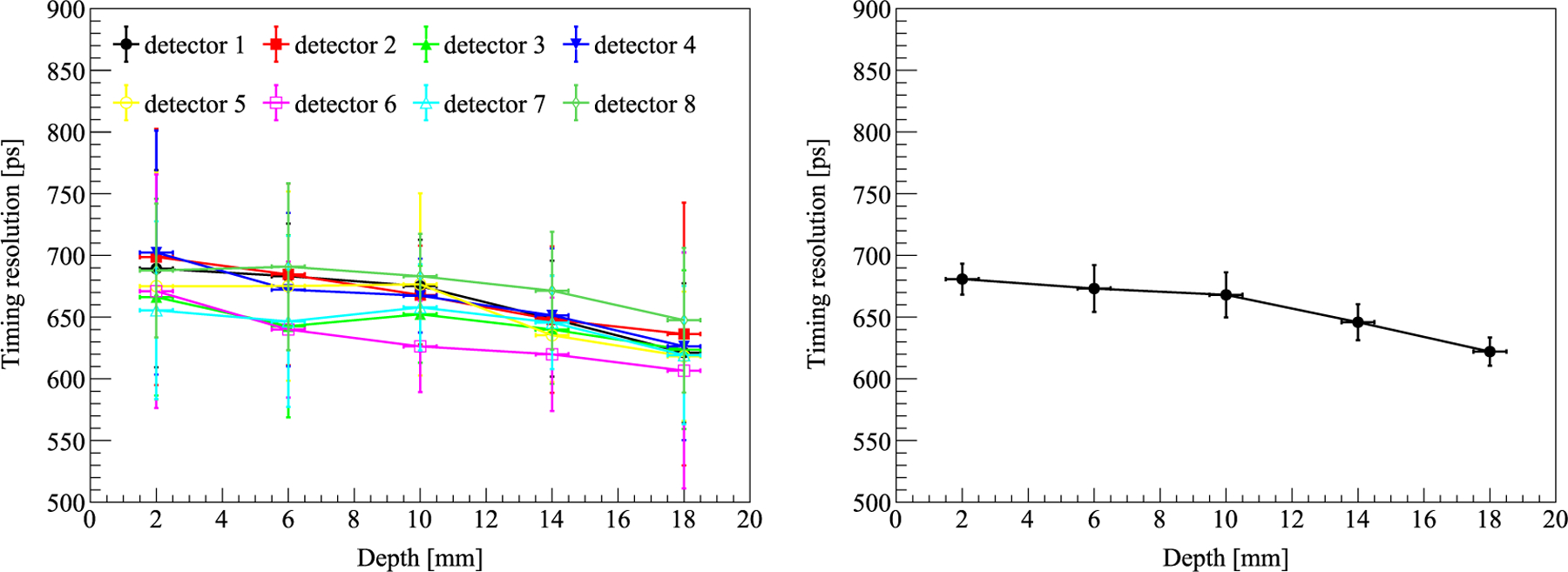
Left: the timing resolution of all 8 LYSO blocks. The error bar is the standard deviation of 16 LYSO units in each detector. Right: the average timing resolution of all 8 LYSO blocks. The error bar is the standard deviation of the 8 LYSO blocks at a specific depth.

The timing resolution of all 8 LYSO blocks is shown in figure 8. The timing resolution got improved when the interaction was far away from the end that was read out by the dual-readout cable, which suggested that the dual-readout cable could slightly delay the timing trigger (either through delay of the cable itself or light traversing the LYSO to reach the side with SiPMs directly connected to the readout). Currently, we have not applied any calibration to consider the effect of this delay. The average timing resolution of all 8 LYSO blocks at all 5 depths was 658.03 ± 15.18 ps.

**Figure 9.**
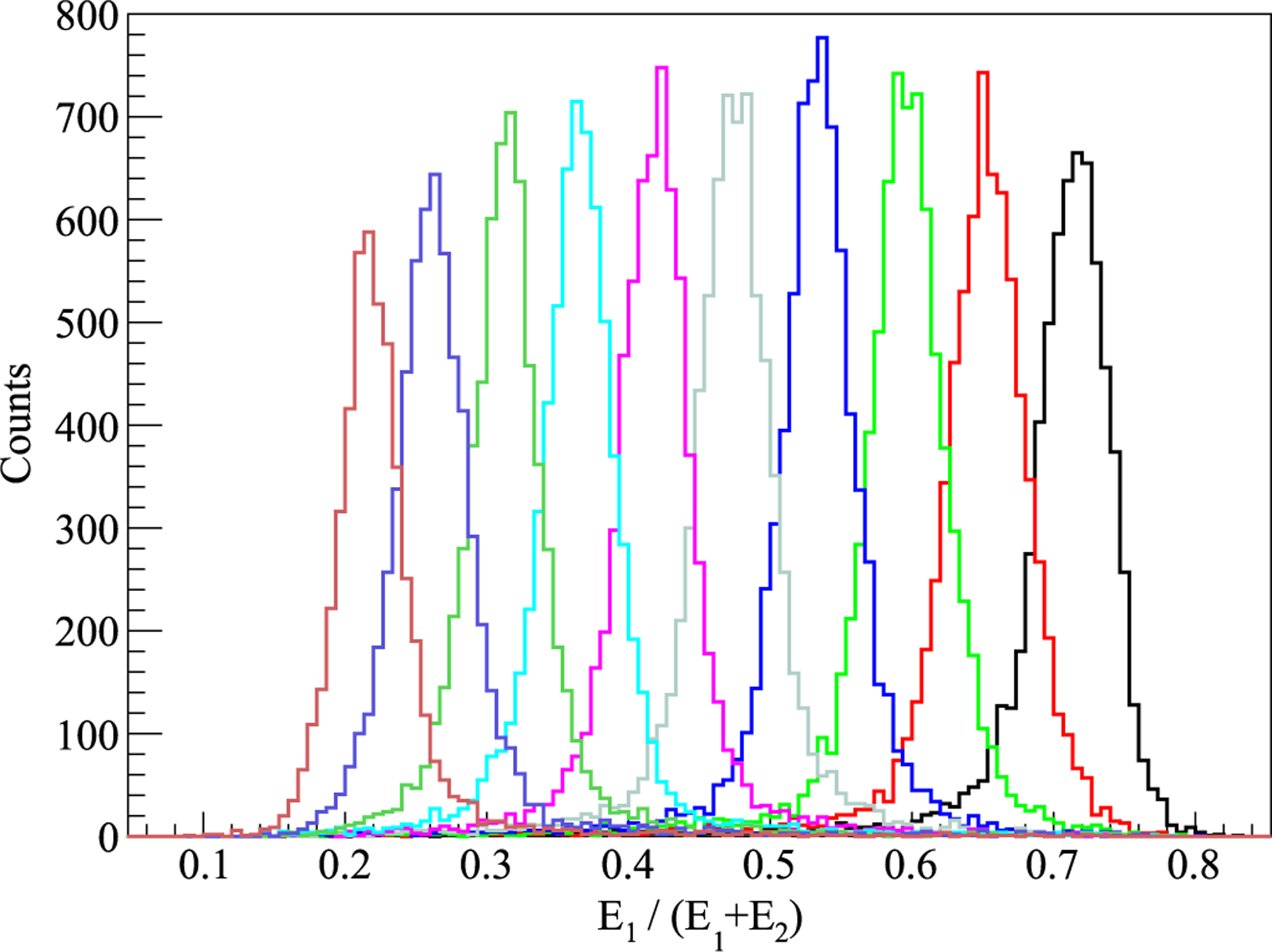
DOI profile of one LYSO unit at 10 depths.

**Figure 10.**
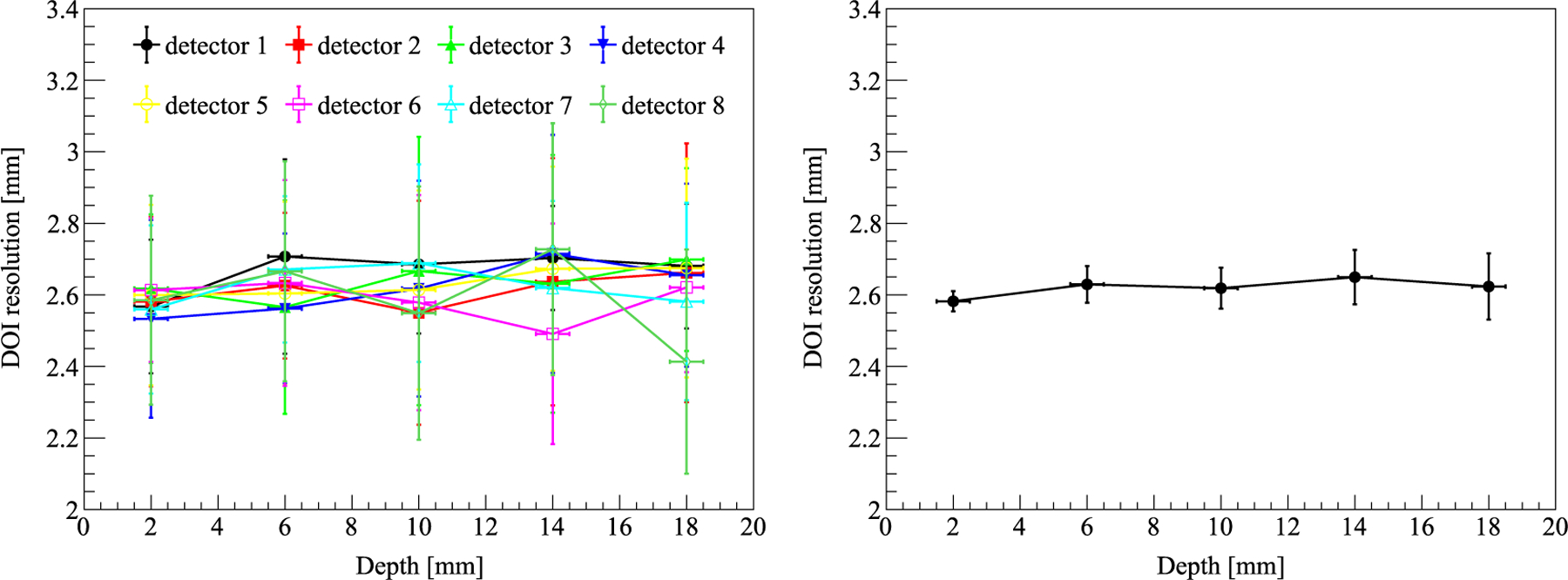
Left: the DOI resolution of all 8 LYSO blocks. The error bar is the standard deviation of 16 LYSO units in each detector. Right: the average DOI resolution of all 8 LYSO blocks. The error bar is the standard deviation of the 8 LYSO blocks at a specific depth.

The DOI ratio histogram of one LYSO unit is shown in figure 9. The DOI resolution of all 8 LYSO blocks is shown in figure 10. The DOI resolution did not show an obvious dependence on the interaction depth, which was aligned with the energy resolution. The average DOI resolution of all 8 LYSO blocks at all 5 depths was 2.62 ± 0.06 mm.

Table 1 summarizes the design and performance of some dual-ended readout detectors for high-resolution PET applications. Compared with our previous direct-couple LYSO blocks detector design, using the dual-readout cables would slightly deteriorate the LYSO blocks detector performance. This is because the dual-readout cables prolong the signal transmission length, which would cause the delay and increase extra signal noise to our detector. However, compared with the other previously published results, our design still can achieve relatively good energy, timing and DOI resolution.

### 3.3. Characteristics of the two detector modules

**Figure 11.**
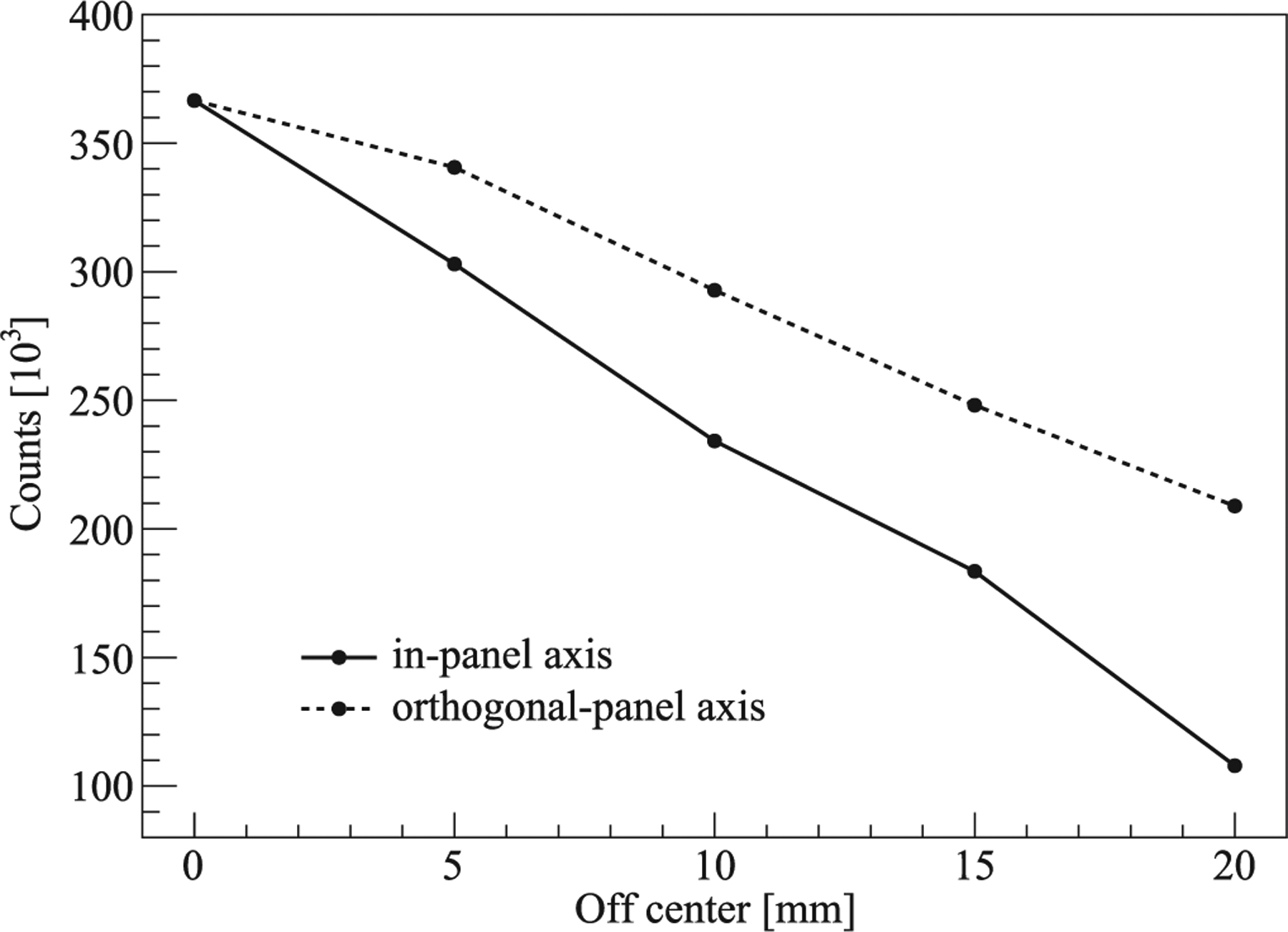
The number of coincidence acquired at different source positions.

The energy and timing resolution of the two detector modules were measured as 16.35% and 0.86 ns, respectively. The number of coincidence acquired at different source positions is shown in figure 11. It can be seen that given the off-center distance, the orthogonal-panel axis has a higher count than the in-panel axis.

**Figure 12.**
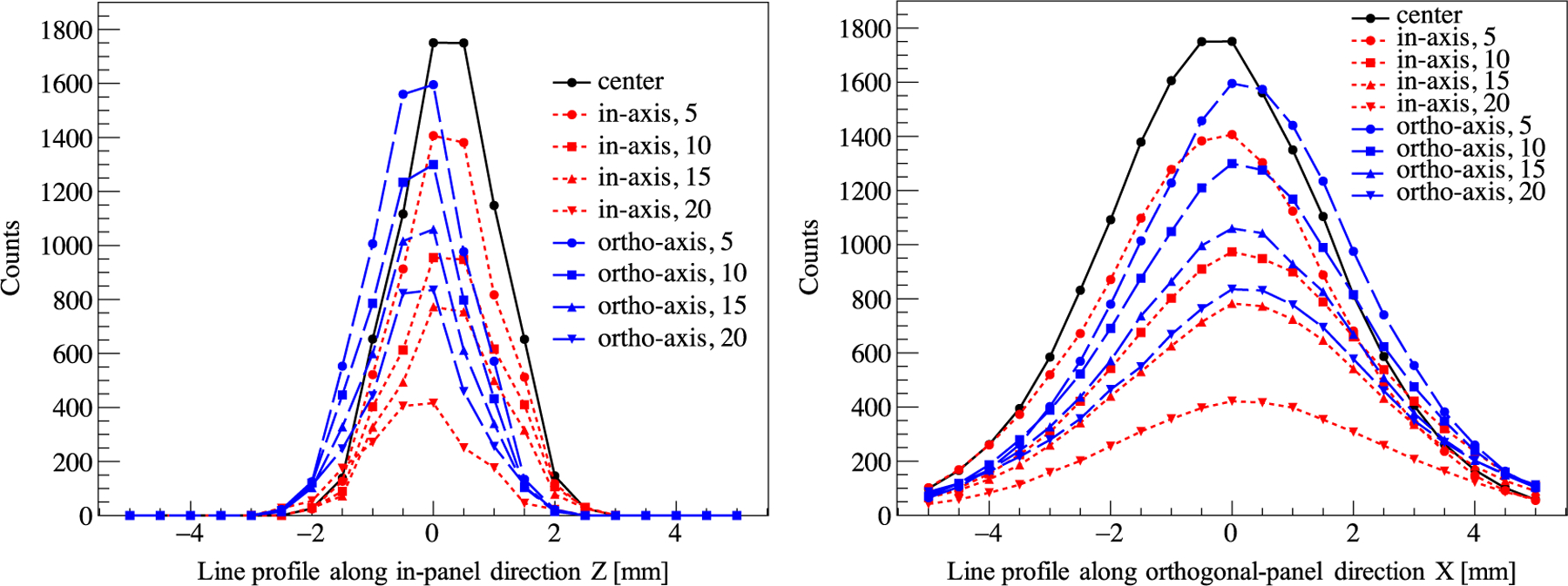
The line profiles of all 9 source positions along the in-panel (left) and orthogonal-panel directions (right). The positive or negative direction of in-panel and orthogonal-panel is shown in figure 1. 0 means the source is at the center of FOV.

Image was reconstructed with the gpurecon program (Cui *et al* 2013), using data collected from a single angle using the setup shown in figure 2. The voxel size was 0.5 × 0.5 × 0.5 mm^3^. The line profiles of all 9 source positions along the in-panel (*z* axis) and orthogonal-panel (*x* axis) directions are shown in figure 12.

**Figure 13.**
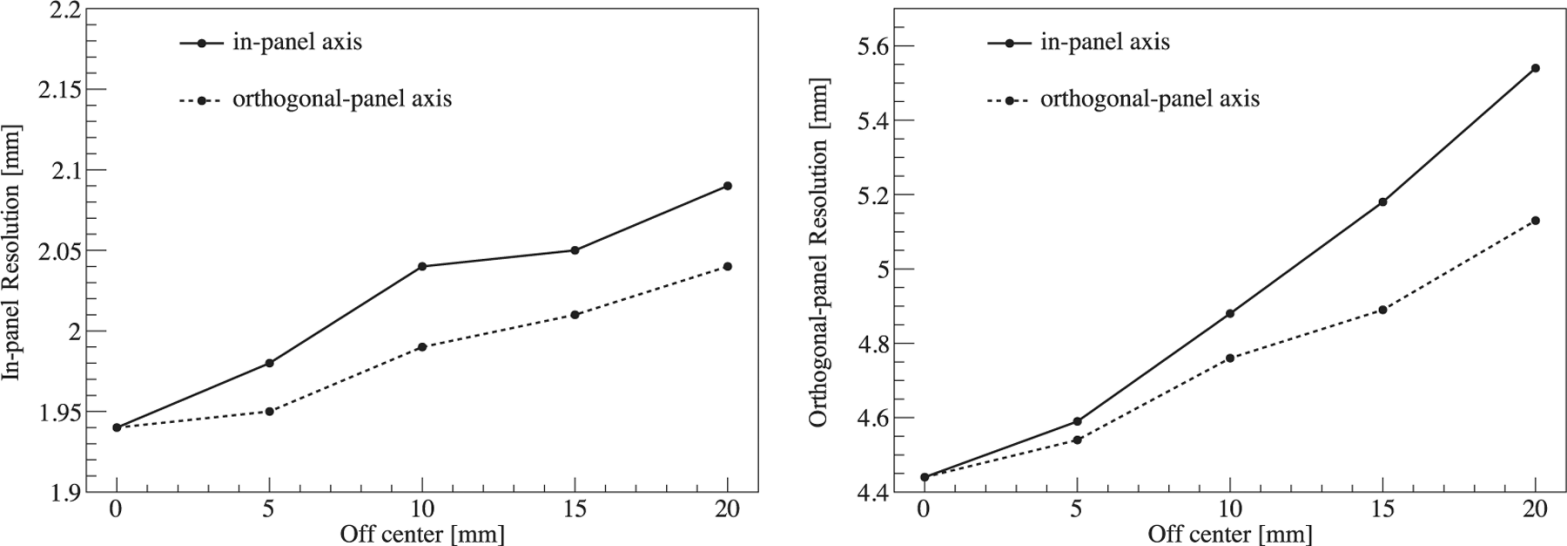
Left: in-panel spatial resolution at different positions within the FOV. Right: orthogonal-panel spatial resolution.

The in-panel and orthogonal-panel spatial resolution were acquired by using a Gaussian function to fit the line profiles, as shown in figure 13. Specifically, the in-panel and orthogonal-panel spatial resolution at the FOV center were 1.94 mm and 4.44 mm, respectively. The results showed that spatial resolution gradually deteriorated when the source was moving away from the FOV center, and the deterioration was worse along the in-panel direction than the orthogonal-panel direction. The difference between the in-panel and the orthogonal-panel spatial resolution was due to the limited angle tomography of the two-panel geometry, which caused the incomplete angular sampling. Specifically, a pair of photons emitted approximately parallel to the panels along the in-panel axis are not likely to be detected, limiting the spatial information along the orthogonal-panel direction and causing the orthogonal-panel spatial resolution to be degraded.

Table 2 summarizes the design and spatial resolution of some breast- and brain-dedicated PET scanners at the FOV center. The comparison showed that this design could achieve a good in-panel spatial resolution, which indicates the potential of utilizing it for the high-resolution PET application.

The comparison of performance in tables 1 and 2 shows that our design system could achieve relatively good energy, timing, DOI resolution, and spatial resolution. Compared with other dual-ended readout PET detectors, our detector has the following advantages:

- We carefully chose the surface roughness of LYSO crystal. The two end surfaces of LYSO crystal were polished to improve the light collection efficiency. The W14 roughness (roughness around 10–14 *μ*m) of lateral surfaces is between the polished surface and the saw-cut surface. This design optimized a good dependency of light collection on depth of interaction with good energy and timing resolution.
- We developed a high-speed dual-readout cable that allows for a compact housing of dual-readout boards in one side of detector blocks with low signal loss.
- We used the TOFPET2 ASICs and PETsys electronics that are commercially available and scalable to high channel density.

## 4. Conclusions

In this work, a dual-ended readout detector module based on SiPM and TOFPET2 ASIC is characterized. The LYSO crystal size was 1 × 1 × 20 mm^3^ and the assembled module size is 53.8 × 57.8 mm^2^. To achieve a compact geometry, the ASICs and readout electronics are placed on the same side of the LYSO block by designing a high speed dual-readout cable. The energy, coincidence timing, and DOI resolution of the LYSO blocks were characterized as 16.13% ± 1.01%, 658.03 ± 15.18 ps and 2.62 ± 0.06 mm FWHM, respectively. Compared with previous direct-couple detector design, using the dual-readout cables would slightly deteriorate the detector performance. With no calibration for the cable delay, the time resolution of the two detector modules (4 LYSO blocks in coincidence with 4 LYSO blocks) was 860 ps. The spatial resolution of two detector modules is characterized by a Na-22 source. The in-panel and orthogonal-panel spatial resolution were measured as 1.9 and 4.4 mm. Compared with previous organ-dedicated PET systems, the sub-scanner shows good in-panel spatial resolution.

## Figures and Tables

**Table 1.** Design and performance of high-resolution dual-ended readout PET detectors.

Citation	Crystal (mm^3^)	Detector Size (mm^3^)	Photo sensor	Energy (%)	Time (ns)	DOI (mm)
This paper	LYSO 1 × 1 × 20	25.8 × 25.8 × 20	SiPM	16.13	0.66	2.62
(Li and Abbaszadeh 2019)	LYSO 1 × 1 × 20	25.8 × 25.8 × 20	SiPM	15.66	0.60	2.33
(Godinez *et al* 2012)	LYSO 1.5 × 1.5 × 20	22 × 22 × 20	PSPMT	19	2.4	2.9
(Kolb *et al* 2014)	LSO 1.55 × 1.55 × 20	19.0 × 19.2 × 20	G-APD	12.8	1.14	2.9
(Shao *et al* 2014)	LYSO 1.9 × 1.9 × 30	16 × 16 × 30	SSPM	17.6	2.8	5.6
(Kuang *et al* 2018)	LYSO 0.97 × 0.97 × 20	11.6 × 11.6 × 20	SiPM	16.7	1.41	2.1
(Du *et al* 2018)	LYSO 0.95 × 0.95 × 20	46 × 46 × 20	SiPM	23.8	1.78	2.81
(Kuang *et al* 2019)	LYSO 0.5 × 0.5 × 20	10 × 10 × 20	SiPM	21	1.23	2.84

**Table 2.** Design and performance of high-resolution dual-ended readout PET detectors.

Citation	Organ	Geometry	Crystal (mm^3^)	Resolution (mm)
This paper	—	Dual panel	1 × 1 × 20	IP 1.9, OP 4.4
(Freifelder *et al* 2001)	Breast	Dual plates	Continuous	IP 3.8, OP 4.6
(Murthy *et al* 2000)	Breast	Dual panel	Continuous	2.8
(Baghaei *et al* 2000)	Breast	Partial ring	2.7 × 2.8 × 19	2.8
(Doshi *et al* 2000)	Breast	Dual panel	3 × 3 × 20	2.3
(Karimian *et al* 2005)	Breast	Ring	3 × 5 × 20	radial 2.8, axial 3.8
(Watanabe *et al* 2002)	Brain	Ring	2.8 × 6.6 × 30	radial 2.9, axial 2.9
(Yoshida *et al* 2006)	Brain	Ring	2.9 × 2.9 × 7.5	radial 3.1, axial 3.1
(Yamamoto *et al* 2011)	Brain	Ring	4.9 × 5.9 × 7	radial 4.0, axial 3.5
(Bauer *et al* 2016)	Brain	Ring	1.5 × 1.5 × 10	radial 2.8, axial 2.0
(Wienhard *et al* 2002)	Brain	Octagon	2.1 × 2.1 × 7.5	2.4
(Tashima *et al* 2015)	Brain	Helmet-chin	2.8 × 2.8 × 7.5	3.0

IP: in-panel. OP: orthogonal-panel.
